# Congenital lactose intolerance is triggered by severe mutations on both alleles of the lactase gene

**DOI:** 10.1186/s12876-015-0261-y

**Published:** 2015-03-21

**Authors:** Lena Diekmann, Katrin Pfeiffer, Hassan Y Naim

**Affiliations:** Department of Physiological Chemistry, University of Veterinary Medicine Hannover, Buenteweg 17, D-30559 Hannover, Germany

**Keywords:** Carbohydrate malabsorption, Lactase deficiency, Brush border membrane enzymes, Compound heterozygote inheritance

## Abstract

**Background:**

Congenital lactase deficiency (CLD) is a rare severe autosomal recessive disorder, with symptoms like watery diarrhea, meteorism and malnutrition, which start a few days after birth by the onset of nursing. The most common rationales identified for this disorder are missense mutations or premature stop codons in the coding region of the lactase-phlorizin hydrolase (LPH) gene. Recently, two heterozygous mutations, c.4419C > G (p.Y1473X) in exon 10 and c.5387delA (p.D1796fs) in exon 16, have been identified within the coding region of LPH in a Japanese infant with CLD.

**Methods:**

Here, we investigate the influence of these mutations on the structure, biosynthesis and function of LPH. Therefore the mutant genes were transiently expressed in COS-1 cells.

**Results:**

We show that both mutant proteins are mannose-rich glycosylated proteins that are not capable of exiting the endoplasmic reticulum. These mutant proteins are misfolded and turnover studies show that they are ultimately degraded. The enzymatic activities of these mutant forms are not detectable, despite the presence of lactase and phlorizin active sites in the polypeptide backbone of LPH-D1796fs and LPH-Y1473X respectively. Interestingly, wild type LPH retains its complete enzymatic activity and intracellular transport competence in the presence of the pathogenic mutants suggesting that heterozygote carriers presumably do not show symptoms related to CLD.

**Conclusions:**

Our study strongly suggests that the onset of severe forms of CLD is elicited by mutations in the LPH gene that occur in either a compound heterozygous or homozygous pattern of inheritance.

## Background

Congenital lactase deficiency (CLD, OMIM 223000) is a rare and severe autosomal recessive disorder, affecting newborn infants [1-7]. Symptoms of CLD, like watery diarrhea, meteorism and malnutrition, begin a few days after birth by the onset of nursing. Small intestinal biopsy specimens reveal normal shape of the microvilli, but very low levels of lactase-phlorizin hydrolase (LPH) [NP_002290], the enzyme responsible for the digestion of lactose, the main carbohydrate in mammalian milk [8,9]. The treatment strategy in those cases is the removal of lactose from the diet, but this has to occur extremely rapidly due to the life-threatening dehydration and electrolyte loss in the newborns.

LPH is a type I transmembrane glycoprotein that is composed of 4 homologous protein domains, which reveal 38% to 55% identity to each other [10]. Due to the high similarity among the four domains, the consensus has emerged that LPH has arisen from two subsequent gene duplications [11]. Out of these, only domain III with the phlorizin-hydrolase activity at position Glu1273 and domain IV with the lactase activity at Glu1749 are present in mature LPH [12]. LPH is synthesized as a polypeptide precursor that co-translationally acquires N-linked glycans in the ER and forms homodimeric quaternary structure before getting trafficked to the Golgi apparatus. In addition to N-glycosylation, LPH is also O-glycosylated. Both types of glycosylation are crucial for the correct folding, proper protein trafficking and subsequent enzymatic activity. Thus, altered glycosylation can affect the activity as well as the cell surface exposure of LPH [13,14]. Also, homodimerization of LPH has been shown to be required for the proper activity of this protein [15,16].

Appearance of premature stop codons and therefore truncated protein as a result of frame shifts, missense mutations in the coding region of LPH or exon duplication are the most common rationales identified so far for CLD [3,6,17]. Some other cases include mutations leading to single amino acid substitutions that can interfere with the proper maturation and function of LPH [4,5]. Nevertheless, few studies until present have related the genetics in CLD with the function of the mutations and their implications in the onset of the disease. Recently, two heterozygous mutations have been identified within the coding region of the lactase gene in a Japanese infant with CLD, both resulting in a truncated protein [3]. In this study we investigated the two mutations, Y1473X and D1796fs at the biochemical and cellular levels, by analyzing the biosynthetic and functional features of mutated LPH and examined their potential influence on the wild type LPH, mimicking thus the situation in heterozygote carriers of these mutations.

## Methods

### Materials

Tissue culture dishes were obtained by Sarstedt (Germany). Dulbecco’s modified Eagle’s medium (DMEM, 1.0 g/L Glucose), minimum essential medium (MEM), chloroquine, pepstatin, leupeptin, antipain, aprotinin, trypsin-inhibitor, phenylmethanesulfonyl fluoride, Triton X-100 and Protein-A Sepharose were purchased from Sigma (Germany). Trypsin-EDTA, streptomycin, penicillin, glutamine and fetal calf serum (FCS) were acquired from PAA Laboratories GmbH (Germany). DEAE-dextran was purchased from Pharmacia (Germany). Proof reading Isis DNA polymerase was obtained from MP Biomedicals. Molecular weight standard for SDS-PAGE was purchased from Thermo Scientific GmbH (Germany). L-[^35^S] methionine was acquired from Perkin Elmer. Restriction enzymes were obtained from Thermo Scientific GmbH (Germany). Glucose oxidase-peroxidase mono-reagent for detection of glucose was purchased from Axiom (Germany) and endoglycosidase H was from Roche Diagnostics (Germany). DTT was purchased from Carl Roth GmbH (Germany).

### Antibodies

Monoclonal mouse antibodies MLac1, MLac10 [18] and HBB 1/909 [19] were used against human intestinal LPH. For immunoprecipitation a mixture of these antibodies was used to detect all conformations and glycoforms of LPH, whereas for immunofluorescent studies HBB 1/909 was used. The secondary antibody coupled to Alexa Fluor 488 dye was obtained from Invitrogen (Germany).

### Generation of complementary DNA clones

LPH was cloned in the vector pSG5 with the *Eco* RI restriction sites of LPH [20]. The mutations were introduced into the cDNA of LPH by mutagenesis-PCR using the following primers:

- LPH-Y1473X: 5′-TGAAGCGGGCCTGAACTACTAGTTGAGGCTCATCG-3′ and 3′-CGATGAGCCTCAACTAGTAGTTCAGGCCCGCTTCA-5′
- LPH-D1796fs: 5′-ACACAGTTTGGAGTGCCATGGCAATTTTGAGTGGGCC-3′ and 3′-GGCCCACTCAAAATTGCCATGGCACTCCAAACTGTGT-5′

Oligonucleotides were provided by Sigma (Germany) and the sequence analysis was performed by GATC Biotech AG (Germany).

### Transient transfection of COS-1 cells, biosynthetic labeling, immunoprecipitation and enzymatic activity measurement

COS-1 cells were cultured in humidified atmosphere containing 5% CO_2_ at 37°C in Dulbecco’s Modified Eagle’s Medium (DMEM) containing 10% FCS and 5% penicillin and streptomycin. Cells were seeded at a confluency of 30–40% and then transfected with 5 μg of cDNA encoding wild type LPH or LPH-Y1473X and LPH-D1796fs by the diethylaminoethyl-dextran method [20]. Metabolic labeling was performed with 40 μCi [^35^S] methionine for 6 h continuously or in a pulse chase experiment for different time points in methionine-free MEM medium. Cells lysis and immunoprecipitation with a mixture of monoclonal anti-LPH antibodies conjugated to Protein-A-sepharose were performed as described before [17,19]. The immunoprecipitates were either treated with endo H for 90 min at 37°C, as previously described [21] to examine the glycosylation pattern or incubated with lactose (28 mmol/ L) for 1 h at 37°C to determine the lactase activity according to Dahlqvist using lactose as the substrate [22]. The amount of glucose generated by lactose hydrolysis was assessed by the Glucose oxidase-peroxidase mono-reagent method. Finally, the samples were analyzed using a 6% SDS-PAGE according to Laemmli (1970) and the protein bands were detected by a phosphorimaging device and quantified using the Quantity One® software from Bio Rad Laboratories GmbH (Germany).

### Confocal fluorescence microscopy

Transiently transfected COS-1 cells, expressing the wild type LPH, LPH-Y1473X and LPH-D1796fs, were grown on cover slips, fixed with 4% Paraformaldehyde and permeabilized with 0.5% Saponin. Immunolabeling was carried out using anti-LPH antibody HBB 1/909 (1:1000) as the primary antibody and anti-mouse IgG conjugated with Alexa 488 (1:500) as the secondary antibody. Confocal laser microscopy was performed with the Leica TCS SP5 microscope using the x63 oil planachromat lens (Leica Microsystems, Germany).

## Results and discussion

### Biosynthesis, processing, cellular localization and function of the mutants LPH-Y1473X and LPH-D1796fs

Until present seven mutations in the coding region of LPH have been identified in Finnish families. Recent genetic testing has revealed two novel mutations, Y1473X and D1796fs, which were found as compound heterozygous pattern in a Japanese infant with CLD [3]. The genotype/phenotype relationship is examined in this paper at the biochemical and cell biological levels. Both mutations are located in domain IV of the extracellular region of LPH compatible with partial truncation of homologous domain IV and complete elimination of the entire membrane anchor as well as the cytoplasmic tail.

Since both mutations appeared in a compound heterozygous mode in one patient, we were interested in investigating the influence of each single mutation *per se* on the enzymatic function and intracellular transport events of LPH.

**Figure 1:**
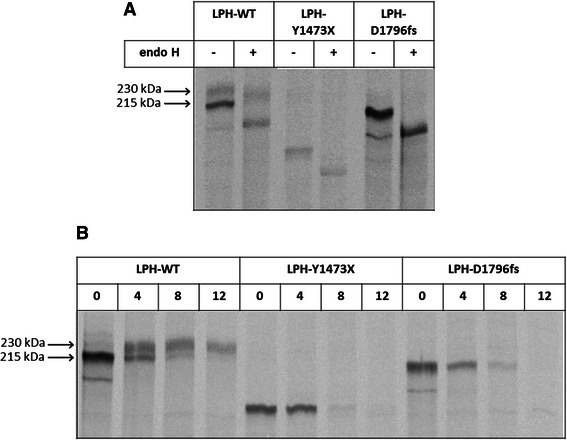
**Biosynthesis and transport kinetics of wild type and mutant proteins.** COS-1 cells were transiently transfected with cDNAs encoding the wild type LPH, LPH-Y1473X or LPH-D1796fs and used 48 h after transfection. **A)** The cells were metabolically labeled with [^35^S] methionine for 6 h continuously, lysed, immunoprecipitated with mAb anti-LPH and treated with endo H. **B)** The cells were labeled with [^35^S] methionine for 2 h and chased for 0, 4, 8 and 12 h, followed by cell lysis and immunoprecipitation. In all these experiments, the proteins were subjected to SDS-PAGE and autoradiography. The quantification was performed by Quantity One® software.

For this purpose the mutations, Y1473X and D1796fs, were introduced into the coding region of wild type LPH separately (thereafter referred to as LPH-Y1473X and LPH-D1796fs) and the generated mutants were expressed in COS-1 cells. Detergent extracts of the biosynthetically-labeled transfected cells were immunoprecipitated. To assess the differences in maturation state and glycosylation pattern among wild type LPH, LPH-Y1473X and LPH-D1796fs, the immunoprecipitates were treated with endoglycosidase H [21]. Endo H cleaves exclusively mannose-rich and some hybrid types of N-glycans which exist in the ER up to *cis*-Golgi. Figure 1A shows that wild type LPH revealed a 215 kDa mannose-rich glycosylated, endo H-sensitive band and a 230 kDa endo H-resistant complex glycosylated band. By contrast, LPH-Y1473X and LPH-D1796fs revealed exclusively endo H-sensitive mannose-rich protein bands respectively smaller than their untreated forms. We further addressed the trafficking of the two mutant proteins by performing pulse chase experiments. For this purpose, COS-1 cells were biosynthetically labeled for 2 h and chased for 0, 4, 8 and 12 h. Figure 1B shows that wild type LPH reached the status where 50% of the protein is mannose-rich glycosylated and 50% is complex glycosylated after 4 h of chase. After 12 h of chase, only the mature form was detectable. The mutated protein variants, LPH-Y1473X and LPH-D1796fs, persisted as mannose-rich glycosylated proteins, but remarkably the bands disappeared almost completely after 8 h of chase. To examine the possibility whether the mutants are ultimately secreted, the media of the biosynthetically labeled COS-1 cells were collected and immunoprecipitated. However, neither the wild type LPH nor the two mutated proteins exhibited any secreted forms (data not shown). These results indicate clearly that the mutants persist in their mannose-rich glycosylated forms, likely in the ER, and are ultimately degraded.

**Figure 2:**
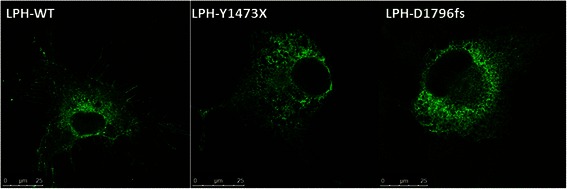
**Intracellular localization of wild type and the mutants in COS-1 cells.** For visualization of LPH, transiently transfected COS-1 cells, expressing the wild type LPH, LPH-Y1473X or LPH-D1796fs, were fixed with 4% paraformaldehyde and permeabilised with 0.5% saponin. Immunolabeling was carried out using mAb anti-LPH (1:1000) as the primary antibody and anti-mouse IgG conjugated with Alexa 488 as the secondary antibody (1:500). The samples were analyzed by confocal laser microscopy.

Persistence of the two mutants as mannose-rich glycosylated species is suggestive of an ER localization of the two protein variants. To substantiate these results using a different procedure, we analyzed the intracellular localization of the mutants by confocal immunofluorescence microscopy. As shown in Figure 2, wild type LPH is located in the ER and the Golgi apparatus as well as at the cell surface, while both mutant variants were predominantly located in the ER as assessed by the typical ER net-like structures.

The lactase active site is located at Glu1749, while that of phlorizin-hydrolase at Glu1273 [12]. The mutant LPH-Y1473X lacks the lactase activity site and it was therefore expected that this mutant reveals no lactase activity, which we confirmed. The mutant LPH-D1796fs contains the lactase active site, but its activity towards lactose was undetectable (data not shown). These results indicate that the compound heterozygote pattern of these two functional-inactive mutants is responsible for complete lactose intolerance in the infant.

### Potential effects of the mutants LPH-Y1473X and LPH-D1796fs on wild type LPH in a heterozygote background

CLD in the patient was initially suggested by typical malabsorption symptoms upon milk uptake and subsequently through the identification of the mutations Y1473X and D1796fs on each allele of the LPH gene.

**Figure 3:**
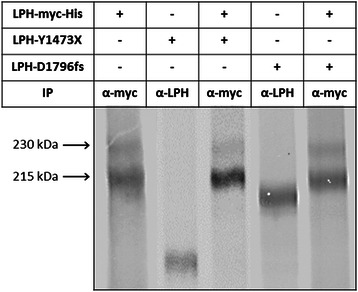
**Potential interaction of LPH-Y1473X and LPH-D1796fs with wild type LPH in co-transfection experiments.** COS-1 cells were transiently transfected either with cDNA encoding wild type LPH tagged with myc (LPH-myc) or cDNA clones encoding the mutants LPH-Y1473X and LPH-D1796fs. In another set of experiments, coexpression of LPH-myc with LPH-Y1473X or with LPH-D1796fs was performed in COS-1 cells. 48 h posttransfection the cells were metabolically labeled with [^35^S] methionine for 6 h continuously, lysed and the lysates were immunoprecipitated with either an antibody against myc to detect wild type LPH or a mixture of mAb anti-LPH antibodies to detect the mutants. Lactase activity was measured by determining the concentration of glucose generated by lactose hydrolysis by the Glucose oxidase-peroxidase mono-reagent method using photometric analysis. The total protein amount of each sample was determined by SDS-PAGE and autoradiography to calculate subsequently the relative specific activity. The quantification was performed by Quantity One® software. The results of LPH-Y1473X were taken from another gel with higher quality.

The compound heterozygote pattern of inheritance in this CLD case raises questions related to potential lactose tolerance in the parents, who are heterozygote carriers of one of the defective LPH alleles. We addressed therefore the question whether a single normal parental allele in conjunction with the diseased one is sufficient to produce lactase protein that has adequate digestive capacity towards dietary lactose. The rationale for this assumption is the dimeric quaternary structure of wild type LPH that is generated in the ER and warrants enzymatic activity as well as transport competence of LPH [16]. Provided that minimal folding requirements are fulfilled, such as correct folding of domains involved in dimerization of LPH, it should be still considered that two different protein isoforms, such as a wild type LPH and a truncated LPH mutant can form heterodimers, which may regulate the enzymatic function. We mimicked therefore the in vivo situation by co-expression of each mutant separately with the wild type LPH, assessed their potential interaction in co-immunoprecipitation experiments and analyzed the activities of the immunoprecipitated LPH. Figure 3 shows protein bands corresponding to wild type LPH that were obtained by immunoprecipitation of cellular lysates from co-transfected and biosynthetically-labelled cells. Since neither the LPH-Y1473X nor LPH-D1796fs mutants appeared in the same electrophoretic lane we conclude that these mutant forms did not co-immunoprecipitate or interact with the wild type LPH.

The enzymatic activities of LPH in the co-transfected experiments did not change or support the notion that interaction has taken place. This in turn clearly indicated that wild type LPH retained its full activity since it has generated its enzymatically active homodimers.

### Mutant LPH-Y1473X or LPH-D1796fs are not temperature-sensitive

**Figure 4:**
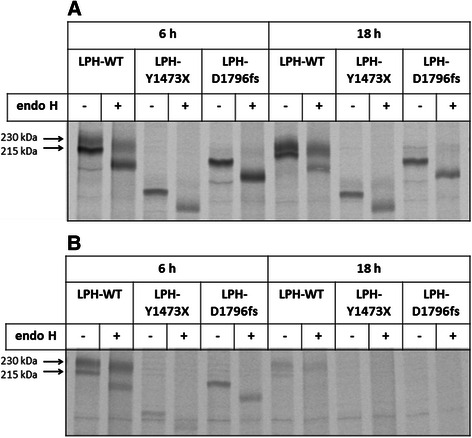
**Biosynthesis and glycosylation pattern of wild type LPH, LPH-Y1473X and LPH-D1796fs at 20°C and 37°C.** Transiently transfected COS-1 cells, expressing wild type LPH, LPH-Y1473X or LPH-D1796fs were labeled with [^35^S] methionine for 3 h and chased for 6 h and 18 h at 20°C **(A)** and 37°C **(B)** followed by cell lysis, immunoprecipitation and endo H treatment. The proteins were subjected to SDS-PAGE and autoradiography. The quantification was performed by Quantity One® software.

Several mutations in proteins that are associated with protein folding diseases exhibit a temperature sensitive pattern, in which lowering the temperature leads to a recovery of correct folding and further protein trafficking out of the ER [5,23-25]. To determine if the mutants are temperature-sensitive and can exit the ER to the Golgi apparatus in a time-delayed manner, a pulse chase experiment was performed at 20°C. The control utilized a similar protocol except that the temperature was changed to 37°C. The wild type LPH was detectable as a mannose-rich glycosylated and a complex glycosylated protein band after 6 h and 18 h of chase at 20°C incubation temperature (Figure 4). At this temperature proteins are predominantly blocked in the Golgi apparatus [26]. This becomes evident in the 18 h chase time point, which shows substantial increase in the intensity of the complex glycosylated mature protein that is processed in the Golgi apparatus. In contrast to the wild type LPH, both mutant proteins appeared as mannose-rich glycosylated species after 6 h of chase at 20°C and did not reach a further maturation status after 18 h of chase, since no complex glycosylated band appeared. The same experiment was performed at 37°C. Interestingly, wild type LPH was still detectable after 18 h of chase, mainly as the mature complex glycosylated form, but LPH-Y1473X or LPH-D1796fs disappeared completely suggesting that the mutants are subject to a degradation mechanism in the ER.

## Conclusions

The hydrolysis of lactose, the main carbohydrate in mammalian milk in the intestinal lumen is achieved by LPH that cleaves the β-glycosidic linkage between glucose and galactose and enables the transport of the liberated monosaccharides across the brush border membrane of intestinal epithelial cells into the cell interior. The failure to digest a disaccharide to its fundamental monosaccharides, for example due to the absence of the corresponding disaccharidase, results in carbohydrate malabsorption. One type of carbohydrate malabsorption is congenital lactase deficiency (CLD), a rare and severe autosomal recessive gastrointestinal disorder, initially affecting newborns [1,6]. Typical symptoms of CLD start from a few days after birth, consisting of liquid and acidic diarrhea, meteorism and severe malnutrition. Appearance of premature stop codons leading to an early truncation of the protein or missense mutations in the coding region of LPH are the common causes identified so far for this disease [1,2,6].

Recently, two novel mutations in the gene of LPH, leading to an early truncation due to a stop codon and a frame shift, were found in a compound heterozygous mode in a Japanese infant with CLD [3]. In this study we showed that these mutants persist predominantly as a mannose-rich glycosylated protein in the ER, are enzymatically inactive and thereby trigger the onset of CLD. Furthermore, our data suggest no interaction between these mutants to generate pseudo-heterodimer complexes that may alter the functional or trafficking characteristics of either one of them. Heterodimeric interactions have been described for many proteins resulting in functional complexes such as connexin heterodimers, G-protein-coupled receptors or GABA(B) receptors [27-29]. An interaction between the wild type LPH and either one of the mutants does not occur and lead to variations in the enzymatic function of LPH. These data explain why the parents do not reveal symptoms associated with lactose intolerance.

The mutations described in this report are similar to another nonsense mutation (Y1390X), called Fin(major), which was detected with 84% occurrence in a study with 32 Finnish CLD patients [6]. This mutated protein is also enzymatically inactive and is retained in the ER.

In conclusion, our study suggests that the onset of severe osmotic diarrhea due to CLD is elicited by severe mutations in the LPH gene that occur in either a compound heterozygous or homozygous pattern of inheritance. This view is supported by the observations that a heterozygote wild type LPH retains its complete activity and intracellular maturation in presence of a pathogenic LPH mutant as shown in co-expression experiments. Wild type LPH generates normally homodimers that are enzymatically active and transport competent. Our study suggests that an interaction between a wild type LPH monomer with a pathogenic LPH mutant apparently does not exist, otherwise heterodimers with altered function, trafficking and maturation characteristics of LPH would have been generated. Nevertheless, additional studies are required with a panel of LPH mutants with variations in their folding pattern, function and pathogenicities to determine whether a potential interaction between these mutants and wild type LPH occurs, which would be associated with mild to severe forms of CLD.

## Abbreviations

CLD: Congenital lactase deficiency
LPH: Lactase-phlorizin hydrolase
ER: Endoplasmic reticulum
Endo H: Endoglycosidase H
